# Longitudinal Hammersmith Infant Neurological Examination (HINE) Trajectories in Children with Cerebral Palsy Identified in High-Risk Follow-Up

**DOI:** 10.3390/jcm14051572

**Published:** 2025-02-26

**Authors:** Vera Joanna Burton, Sujatha Kannan, Srishti Jayakumar, Gwendolyn Gerner, Salome West, Gayane Yenokyan, Andrea F. Duncan

**Affiliations:** 1 Department of Neurology and Developmental Medicine, Kennedy Krieger Institute, Baltimore, MD 21205, USA; 2 Division of Neurology and Neurosurgery, The Johns Hopkins School of Medicine, Baltimore, MD 21224, USA; 3 Department of Pediatrics, The Johns Hopkins School of Medicine, Baltimore, MD 21224, USA; skannan3@jhmi.edu (S.K.); srishtijayakumar@jhu.edu (S.J.); 4 Department of Anesthesia and Critical Care Medicine, The Johns Hopkins School of Medicine, Baltimore, MD 21224, USA; 5 Division of Neonatology, The Johns Hopkins School of Medicine, Baltimore, MD 21224, USA; 6 Department of Neuropsychology, Kennedy Krieger Institute, Baltimore, MD 21205, USA; gernerg@kennedykrieger.org; 7 Division of Psychiatry and Behavioral Sciences, The Johns Hopkins School of Medicine, Baltimore, MD 21224, USA; 8 Department of Biostatistics, Johns Hopkins Bloomberg School of Public Health, Baltimore, MD 21205, USA; 9 Department of Pediatrics, University of Pennsylvania Perelman School of Medicine, Philadelphia, PA 19104, USA; 10 Department of Pediatrics, Division of Neonatology, Children’s Hospital of Philadelphia, Philadelphia, PA 19104, USA

**Keywords:** cerebral palsy, early diagnosis, HINE, longitudinal evaluation

## Abstract

**Background/Objectives**: The Hammersmith Infant Neurological Examination (HINE) is a standardized neurologic exam for infants between 2 and 24 months. Scores can be compared to optimality cutoffs as one component to support an early diagnosis of cerebral palsy (CP). Some prognosis is also possible for infants diagnosed with CP. We aimed to understand the longitudinal trajectories of HINE scores in infants who were ultimately diagnosed with CP. **Methods**: Clinical records were reviewed for children who were diagnosed with CP in two high-risk infant follow-up clinics with HINE scores from at least two visits between the corrected ages of 3 months and 2 years. Trajectories were calculated individually and by group for infants in four categories—term neonatal hypoxic ischemic encephalopathy (HIE), term perinatal arterial ischemic stroke (PAIS), premature infants with brain injury, and “Other” (term infants with congenital malformations and/or congenital hydrocephalus). The changes in HINE scores between clinic visits were compared using linear mixed-effect models with a random intercept, pulling data by diagnostic group across visits and accounting for within-child correlations of scores over the follow-up time. **Results**: The changes in HINE scores for sixty children (twenty-five with prematurity, eighteen with HIE, seven with PAIS, and ten in the other category) were assessed. The linear mixed-effect models indicated that the infants with PAIS had an estimated 10.8-point increase in total HINE scores after 9 months of age compared to earlier assessments (95% CI [2.5, 19.2]. There was no statistically significant improvement in the scores among the infants in the other brain injury groups. The infants with PAIS had an estimated 2.9-point increase in HINE asymmetry scores after 9 months of age compared to prior visits (95% CI [0.7, 5.1]). None of the other diagnostic categories had statistically significant increases in asymmetry scores over time. **Conclusions**: The children with PAIS with resultant hemiplegia showed increasing HINE scores throughout the first two years of life. In contrast, the HINE scores remained stable for those children with term HIE, prematurity-associated brain injury, and congenital malformations and/or congenital hydrocephalus diagnosed with CP. Tracking individual changes (or stability) in HINE scores can aid diagnosis, inform prognosis, and guide the design of clinical trials targeting neurologic injury.

## 1. Introduction

Cerebral palsy (CP) is one of the most common motor disabilities in childhood, affecting roughly 18 million people worldwide [1,2,3,4]. It is defined as a group of permanent disorders of movement and posture caused by perinatal brain injury that result in activity limitations [5]. Advocacy for the early identification of cerebral palsy in order to institute therapeutic treatment during peak periods of neuroplasticity has existed for some time; however, there have been barriers to the adoption of early diagnosis tools. Until recently, many clinicians believed that they should not make a diagnosis before 12 months and in some cases felt that it was necessary to wait until a patient was 3–5 years of age and demonstrated definitive signs of cerebral palsy on traditional evaluation tools. Even with the available diagnostic tools, there continue to be barriers to clinicians universally adopting these tools, which include a lack of specific biomarkers, difficulty defining functional impairment in young infants, as well as uncertainty with topography and classification presentations in infants compared to children [6,7]. These barriers may delay diagnosis. Continued evidence is necessary to improve clinician ease with early diagnosis.

Establishing an early diagnosis of CP is crucial for optimizing outcomes through early targeted interventions and opportunities for appropriate support for families. Guidelines for early diagnosis were established in 2017 and include the use of a standardized neurologic exam, the Hammersmith Infant Neurological Examination (HINE), as one element of diagnosis [8]. There is a growing body of literature demonstrating the feasibility and effectiveness of using the HINE as one component for supporting the clinical diagnosis of CP under the age of 1 year [9,10]. The HINE is a standardized neurologic exam for infants between 2 and 24 months of age that is composed of 26 items that result in a total score of up to 78 as well as a separate asymmetry score. By assessing various aspects of neurological function, including cranial nerves, posture, movement, tone, reflexes, and reactions, the HINE provides clinicians with an understanding of the infant’s neurological status at a single point in time [11]. Total scores falling below the published optimality scores can support early diagnosis between 3 and 18 months of corrected age [8,12,13,14]. Optimality scores are not the same as diagnostic cutoffs because they are designed such that 10% of children who are typically developing have scores below the optimality score. Therefore, in many cases, serial HINE scores can provide additional information when the initial HINE scores are below the optimality cutoffs. The HINE asymmetry score is calculated by adding the number of items where differences are noted between the right and left sides during the exam [15].

At 3–4 months of corrected age, an HINE score below the optimality cutoffs is an integral part of early diagnosis and risk stratification when combined with a risk history, functional motor exam, neuroimaging, and underlying movement patterns using Prechtl’s General Movement Assessment (GMA) at fidgety age [8,16]. After a corrected age of 3–4 months, additional information may be needed to confirm a clinical diagnosis of CP. One suggestion is the use of more than one HINE evaluation at different points in time, noting that continued performance under optimality scores provides additional support for diagnosing CP. Although not precise, some prognosis is also possible given correlations between HINE scores and Gross Motor Function Classification System (GMFCS) scores for infants diagnosed with CP [11]. The aim of this study was to determine the trajectory of the HINE scores in a population of children diagnosed with CP using early diagnosis criteria.

## 2. Materials and Methods

This was a retrospective chart review of data collected for children diagnosed with cerebral palsy as part of a quality improvement project using published early detection guidelines in two high-risk follow-up clinics, associated with level III and level IV NICUs in Maryland and Pennsylvania, between 2019 and 2023. 61 children with CP were identified who had at least 2 HINE scores available between the ages of 3 months and 2 years when corrected for prematurity.

HINE exams were performed by trained clinicians as per clinic guidelines following the guidance notes available at www.mackeith.co.uk/hammersmith-neurological-examinations (accessed on 12 December 2024). HINE total and asymmetry scores were extracted from clinical visits for any of the following time epochs: visit 1 = 3–4 months corrected age, visit 2 = 5–8 months corrected age, visit 3 = 9–11 months corrected age, visit 4 = 12–15 months corrected age, visit 5 = 16–23 months corrected age, and visit 6 24 to 25 months corrected age. Additional demographic and early diagnosis information included risk history, neuroimaging findings, and GMA classifications at writhing (preterm and term age equivalent, including normal, poor repertoire, and cramped synchronized) and fidgety (3–4 months corrected for prematurity, including present, abnormal, or absent) ages were also extracted [16]. Individual HINE trajectories were calculated for each infant. Additionally, average trajectories were calculated by brain injury category. Categories included term neonatal hypoxic ischemic encephalopathy (HIE), term perinatal stroke (PAIS), and premature infants with brain injury. A small group of term infants with congenital malformations and/or congenital hydrocephalus comprised the “Other” category. Changes in HINE scores between clinic visits by brain injury group were compared using linear mixed-effect modeling with a random intercept for infant, pulling the data across visits by diagnostic group and accounting for within-child correlations of scores over time. Due to non-linear changes in scores over time, two models were considered: (1) with change in linear slope at visit 3 (i.e., model with linear spline) and (2) a binary indicator for visit 3 or later vs. before visit 3. Models were compared using Bayesian Information Criterion (BIC), and the most parsimonious model is described in the Results section. Statistical analyses were performed using STATA version 18 (StataCorp, College Station, TX, USA) [17].

## 3. Results

Sixty children were included in this analysis who had at least two visits and a final diagnostic category. Among them, twenty-five (42%) were classified as premature infants, eighteen (30%) had HIE, seven (12%) had PAIS, and ten (17%) were “Other” (Table 1). Five patients born between 34 and 37 weeks were classified as HIE or PAIS since their primary pathology was thought to be related to their brain injury, not their prematurity. Of the twenty-five infants classified as premature, thirteen were less than 28 weeks (extremely preterm), five ranged from 28 to less than 32 weeks (very preterm), two ranged from 32 to less than 34 weeks, and ten ranged from 34 to less than 37 weeks. There were no statistically significant differences in the age of diagnosis across the age groups. There were no differences in classification by GMA in the writhing age or fidgety age across the diagnostic groups, although the majority of the infants did not have GMA results available at either age. Due to the predetermined brain injury categories, there was an expected statistically significant association between the brain injury category and gestational age (*p* < 0.001). Of the twenty-five premature infants, twenty were less than 34 weeks and five were between 34 and 36 weeks gestational age.

**Figure 1:**
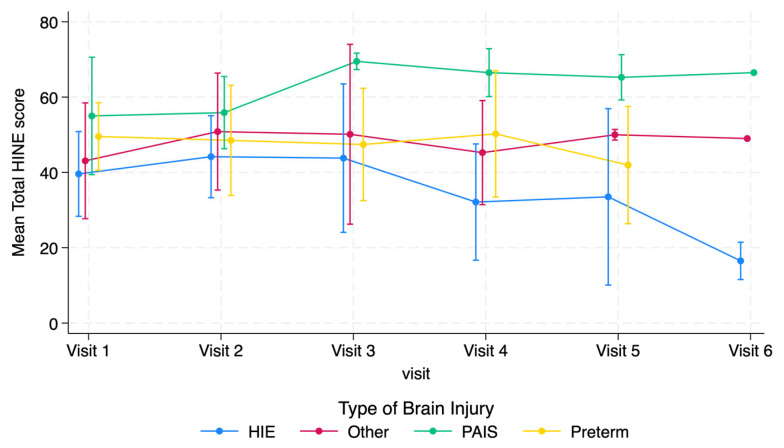
Mean HINE total scores stratified by type of brain injury.

**Figure 2:**
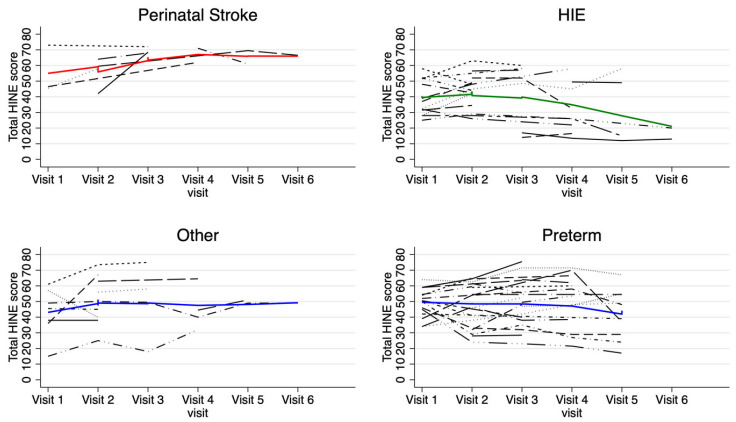
Individual HINE total scores plotted longitudinally. Each black patterned line plots the HINE total score trajectory of a single individual participant. The solid, colored line plots the mean HINE total score trajectory of the whole subgroup.

**Figure 3:**
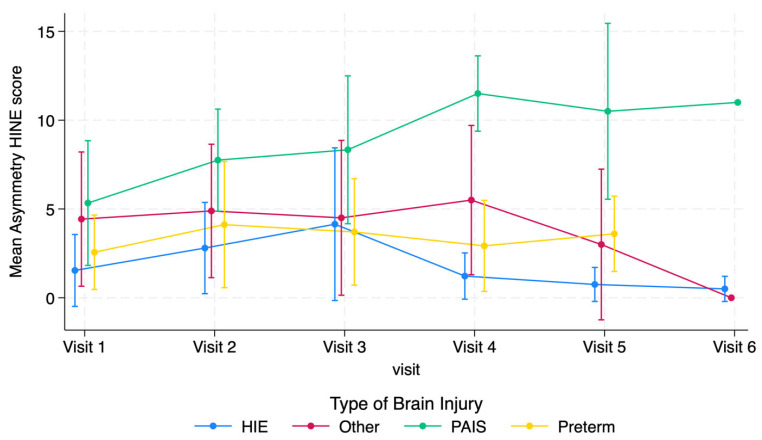
HINE asymmetry scores stratified by type of brain injury.

**Figure 4:**
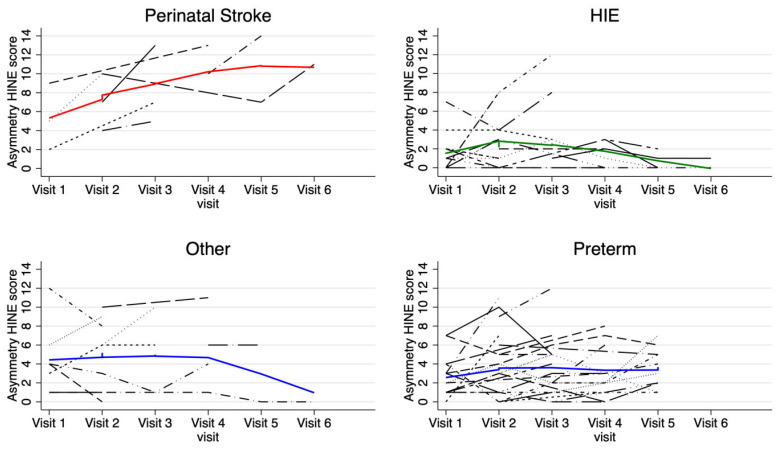
Individual HINE asymmetry scores plotted longitudinally. Each black patterned line plots the HINE asymmetry score trajectory of a single individual participant. The solid, colored line plots the mean HINE asymmetry score trajectory of the whole subgroup.

The individual and average trajectories of the total HINE and asymmetry scores are shown in Figure 1, Figure 2, Figure 3 and Figure 4. The results of linear mixed-effect modeling indicated that the infants with PAIS had an estimated 10.8-point increase in HINE score after 9 months of age (visit 3 and on) compared to prior visits (95% CI [2.5, 19.2]). In contrast, there was no statistically significant change among the infants in any of the other brain injury categories, with a trend toward decreases in scores observed in the infants with HIE, although it did not reach significance due to the small number of children with HIE who had visits 5 and 6.

When the HINE asymmetry scores were analyzed, the infants with PAIS had an estimated 2.9-point increase in asymmetry score after 9 months of age compared to prior visits (95% CI [0.7, 5.1]). No other diagnostic categories demonstrated statistically significant changes in asymmetry scores over time.

## 4. Discussion

This study presents a novel finding that the trajectory of HINE scores in infants with CP with a history of HIE, prematurity, and/or congenital malformations with or without hydrocephalus may remain largely stable over time, without any significant increases in scores, and, in fact, a trend toward decreases in scores was observed in infants with HIE, although it did not reach significance. These results extend the early studies regarding the use of the HINE in children with HIE, which demonstrated neurologic abnormalities in the early weeks for those with severe brain injury that persisted until at least 6 months in children who were diagnosed with CP by 2 years of age [18]. These results also provide further evidence supporting the validity of assessments at 3 to 6 months of corrected age given the stability in the scores for children with CP over time. Specifically, the HINE scores in these early ages can support a clinical diagnosis of cerebral palsy if used in conjunction with other elements, including a risk history, concerning neuroimaging, GMA findings, and functional motor evaluations [8]. A systematic review by Romeo and colleagues [11] demonstrated the excellent sensitivity of the predictive value of the HINE. In the few cases of concerning HINE scores at 3–6 months in infants who did not develop CP, the longitudinal assessments of these infants showed improvements in their exams and HINE scores. Additionally, children born prematurely and followed longitudinally also demonstrated increases in their HINE scores over time [19]. In contrast, the results of this study demonstrate that the HINE scores for these children with CP remained largely stable, unlike the upward trajectory shown in typically developing children [12,19]. Therefore, the improved sensitivity at later ages would appear to be due to scores increasing for typically developing children as the scores for children diagnosed with CP did not show statistically significant increases over time. Thus, in cases where there are not enough other elements of early diagnosis available at a single data point to support a diagnosis of CP, this study provides additional data that serial exams in high-risk neonates, consistent with a diagnosis of CP, show plateauing of scores.

We also note a significant increase in HINE scores after 9 months of corrected age in the cohort of infants with perinatal stroke diagnosed with hemiplegic CP. This finding is consistent with the literature [14] that children with hemiplegic CP sometimes have scores above the optimality cutoffs. Importantly, we found simultaneously that the asymmetry scores significantly increased in this group as well. This supports the emerging data [14] that the asymmetry score, when combined with the total HINE score, has high sensitivity in differentiating infants with hemiplegic CP from typically developing infants.

In addition, these results have important implications for designing clinical trials aimed at very early interventions for CP. Such trials have already begun in other diseases, such as SMA [20]. Knowledge of the expected trajectory of the HINE scores in groups of children at risk for CP provides an opportunity to follow these scores longitudinally as a measure of the efficacy of therapeutic modalities. An improvement in HINE scores in these circumstances can serve as an early indication of treatment efficacy, thereby enabling cost-effective, rapid screening of promising therapies that can be advanced to more extensive long-term trials that are typically time-consuming and expensive. A plateau in scores can also identify infants for whom additional services should be indicated and aid in identifying subpopulations that may be eligible for disease-modifying therapies.

This study has several strengths. The data were obtained from two centers where standardized assessments are conducted in accordance with the early detection guidelines of the Cerebral Palsy Foundation Early Detection & Intervention Network. Given the geographical and compositional heterogeneity of the patient population, these results may have generalizability to other contexts. This study addresses earlier outcomes using the HINE, which is meaningful to providers and families alike. The study does also have limitations. The sample sizes of each diagnostic category are small. Further, since the patients were drawn from a clinical sample, the authors could not control for all the neonatal variables or ensure complete data for each child. Since this database is deidentified, important details reflecting social determinants of health could not be identified from this sample. Further, while the current study does not include a control group of patients at high risk who did not develop CP, this is an important future direction for this work.

## 5. Conclusions

In this clinical cohort from two high-risk follow-up clinics, the children with PAIS with resultant hemiplegia continued to have increasing HINE scores throughout the first two years of life. In contrast, the HINE scores remained stable for premature infants with brain injury and children with HIE diagnosed with cerebral palsy. Understanding changes or lack thereof in individual trajectories will be useful in designing clinical trials that target neurologic injury. Trajectory information may also provide information to aid with prognosis or when serial HINE scores are used as part of diagnosis. Future studies of larger samples with concurrent controls are warranted.

## Figures and Tables

**Table 1:** Demographic information. Demographic and Clinical Characteristics of Infants by Diagnostic Category.

	HIE	Other	PAIS	Preterm	Total	*p*-Value
	N = 18	N = 10	N = 7	N = 25	N = 60	
Sex						0.46
Female	7 (39%)	2 (20%)	3 (43%)	5 (20%)	17 (28%)	
Male	11 (61%)	8 (80%)	4 (57%)	19 (76%)	42 (70%)	
Missing	0 (0%)	0 (0%)	0 (0%)	1 (4%)	1 (2%)	
Gestational Age						<0.001
<28 weeks	0 (0%)	0 (0%)	0 (0%)	13 (52%)	13 (21.7%)	
28–32 weeks	0 (0%)	0 (0%)	0 (0%)	5 (20%)	5 (8.3%)	
32–34 weeks	0 (0%)	0 (0%)	0 (0%)	2 (8%)	2 (3.3%)	
34–37 weeks	3 (17%)	0 (0%)	2 (29%)	5 (20%)	10 (16.7%)	
37–38 weeks	5 (28%)	6 (60%)	2 (29%)	0 (0%)	13 (21.7%)	
39–40 weeks	9 (50%)	4 (40%)	3 (43%)	0 (0%)	16 (26.7%)	
>40 weeks	1 (6%)	0 (0%)	0 (0%)	0 (0%)	1 (1.6%)	
GMA: Writhing						0.60
Normal	0 (0%)	0 (0%)	0 (0%)	0 (0%)	0 (0%)	
poor repertoire	4 (22%)	0 (0%)	1 (14%)	3 (12%)	8 (13%)	
cramped synchronized	0 (0%)	0 (0%)	0 (0%)	2 (8%)	2 (3%)	
Unknown	14 (78%)	10 (100%)	6 (86%)	20 (80%)	50 (83%)	
GMA: Fidget						0.41
Present	1 (6%)	0 (0%)	1 (14%)	2 (8%)	4 (7%)	
Absent	11 (61%)	3 (30%)	2 (29%)	9 (36%)	25 (42%)	
Unknown	6 (33%)	7 (70%)	4 (57%)	14 (56%)	31 (52%)	
CA at diagnosis	7.2 (2.9)	9.6 (6.0)	9.3 (4.6)	8.5 (6.6)	8.4 (5.4)	0.66
AA at diagnosis	6.8 (2.8)	9.4 (5.8)	8.8 (4.7)	8.3 (5.0)	8.1 (4.6)	0.51

GMA: General Movement Assessment; CA: chronological age; AA: adjusted age. Data are presented as mean (SD) for continuous measures and n (%) for categorical measures.

## Data Availability

The raw data supporting the conclusions of this article will be made available by the authors on request.
